# Geographic Distribution and Survival Outcomes for Rural Patients With Cancer Treated in Clinical Trials

**DOI:** 10.1001/jamanetworkopen.2018.1235

**Published:** 2018-08-17

**Authors:** Joseph M. Unger, Anna Moseley, Banu Symington, Mariana Chavez-MacGregor, Scott D. Ramsey, Dawn L. Hershman

**Affiliations:** 1 SWOG Statistics and Data Management Center, Seattle, Washington; 2 Public Health Sciences Division, Fred Hutchinson Cancer Research Center, Seattle, Washington; 3 Sweetwater Regional Cancer Center, Memorial Hospital of Sweetwater County, Rock Springs, Wyoming; 4 Department of Health Services Research, The University of Texas MD Anderson Cancer Center, Houston; 5 Columbia University Medical Center, New York, New York

## Abstract

**Importance:**

Studies showing that patients with cancer from rural areas have worse outcomes than their urban counterparts have relied on cancer population data and did not account for differences in access to care. Clinical trial patients receive protocol-directed care by design, so large clinical trial databases are ideal for examining the impact of rural vs urban residency on outcomes.

**Objective:**

To compare the geographic distribution and survival outcomes for rural vs urban patients with cancer treated in clinical trials.

**Design, Setting, and Participants:**

In this comparative effectiveness retrospective cohort analysis, 36 995 patients from all 50 states enrolled in 44 phase 3 and phase 2/3 SWOG (formerly the Southwest Oncology Group) treatment trials from January 1, 1986, to December 31, 2012, were examined. Seventeen different cancer-specific analysis cohorts were constructed. Data through January 30, 2018, were analyzed.

**Main Outcomes and Measures:**

*Rural* vs *urban* residency was defined using the Rural-Urban Continuum Codes developed by the US Department of Agriculture. Multivariate Cox regression was used to estimate the association of residency with overall survival, progression-free survival, and cancer-specific survival, controlling for major disease-specific prognostic factors and demographic variables and stratifying by study. Different definitions of rurality were examined. The distribution of rural vs urban patients by geographic region was described.

**Results:**

Overall, 27.7% of patients were 65 years or older (range across 17 cohort analyses, 7.8%-74.5%), 40.3% were female in the non-sex-specific analyses (range across 17 cohort analyses, 28.1%-45.9%), and 10.8% were black (range across 17 cohort analyses, 1.9%-22.4%). Overall, 19.4% of patients (7184 of 36 995) were from rural locations. Rural patients were more likely to be aged 65 years or older (rural, 30.7% aged ≥65 years vs urban, 27.0% aged ≥65 years; difference, 3.7%; 95% CI, 2.5%-4.9%; *P* < .001), were less likely to be black (rural, 5.4% vs urban, 12.1%; difference, 6.7%; 95% CI, 6.1%-7.3%; *P* < .001), were similar with respect to sex (rural, 40.4% female vs urban, 39.7% female; difference, 0.6%; 95% CI, −1.4% to 2.6%; *P* = .53), and were well represented within major US geographic regions (West, Midwest, South, and Northeast). Clinical prognostic factors were similar. In multivariable regression, rural patients with adjuvant-stage estrogen receptor–negative and progesterone receptor–negative breast cancer had worse overall survival (hazard ratio, 1.27; 95% CI, 1.06-1.51; *P* = .008) and cancer-specific survival (hazard ratio, 1.26; 95% CI, 1.04-1.52; *P* = .02). No other statistically significant differences for overall, progression-free, or cancer-specific survival were found. Results were consistent regardless of the definition of rurality.

**Conclusions and Relevance:**

Rural and urban patients with uniform access to cancer care through participation in a SWOG clinical trial had similar outcomes. This finding suggests that improving access to uniform treatment strategies for patients with cancer may help resolve the disparity in cancer outcomes between rural and urban patients.

## Key Points

**Question:**

Do rural and urban patients with cancer receiving similar care in clinical trials have similar outcomes?

**Findings:**

In this comparative effectiveness study, 36 995 patients from all 50 states enrolled in 44 SWOG treatment trials from 1986 to 2012, composing 17 different cancer-specific analysis cohorts, were examined. Rural patients had statistically significantly worse survival in only 1 of the 17 analysis cohorts (those with adjuvant-stage, estrogen receptor–negative and progesterone receptor–negative breast cancer) irrespective of how rural residency was defined.

**Meaning:**

Improving access to uniform treatment strategies such as those found in clinical trials may help resolve the disparity in cancer outcomes between rural and urban patients.

## Teaser

This pooled cohort study compares overall, progression-free, and cancer-specific survival of patients living in rural vs urban settings who were enrolled in phase 2 and 3 SWOG treatment trials between 1986 and 2012.

## Introduction

Nineteen percent of the US population overall, and of the US population with cancer in particular, are from rural areas.^1,2^ Rural patients with cancer have been shown to have worse outcomes than their urban counterparts. A major recent report indicated that the age-adjusted rate of cancer deaths in rural areas from 2011 to 2015 was 180.4 per 100 000 individuals, compared with just 157.8 per 100 000 individuals in large metropolitan areas.^2^ Thought leaders have expressed concerns that these differences might be attributed to rural individuals’ reduced access to medical, technological, and financial resources, as well as adverse health status and shortened life spans.^3^ The Cancer Moonshot initiative emphasized that rural patients with cancer experience disproportionate morbidity and mortality and indicated the importance of increased research into disparities between rural and urban patients.^4^ Indeed, disparities in cancer outcomes for rural patients in the United States may actually be increasing rather than decreasing.^2^ Whether this disparity is due to inadequate access to quality cancer care or other characteristics of patients residing in rural areas, such as different clinical, demographic, or disease profiles, is unclear.

In this context, an important question is whether rural and urban patients with cancer who receive similar care have similar outcomes. To address this, we compared survival outcomes between patients with cancer from rural vs urban locales who participated in therapeutic clinical trials. Patients receiving care in this setting are uniformly staged, treated, and followed up under protocol-specific guidelines, reducing the potential influences of inconsistent pretreatment evaluation, care, and posttreatment surveillance. If outcomes between rural and urban patients with cancer in clinical trials are similar, then access to uniform treatment strategies of the type represented by clinical trial care could help alleviate disparities in outcomes.

## Methods

### Patients

Data were derived from patient medical records for trial participants enrolled between January 1, 1986, and December 31, 2012, to clinical treatment trials conducted by SWOG (formerly the Southwest Oncology Group), a National Clinical Trials Network and Community Oncology Research Program group sponsored by the National Cancer Institute (NCI). Only data from phase 3 or large phase 2/3 trials for which the primary analysis was previously published were included. The following cancer types were included: acute myeloid leukemia, brain, breast, colorectal, lung, lymphoma, myeloma, ovarian, prostate, and sarcoma (gastrointestinal stromal tumors). We combined trials with similar histology and stage to increase our power to identify potential differences in survival outcomes between rural and urban patients. In the case of advanced prostate cancer, we analyzed SWOG trials S8894 and S9346 separately, because each of these trials enrolled more than 1000 patients. Each trial included in this analysis was previously approved by an institutional review board; informed consent was previously obtained from all patients for each study included. Institutional review board approval and informed consent of study participants for this comparative effectiveness study was not required because secondary data that were not identifiable were used. The research question and analysis plan were defined prospectively in accordance with the International Society for Pharmacoeconomics and Outcomes Research (ISPOR) reporting guideline^5^ for the conduct of comparative effectiveness studies.

### Covariates and Outcome Variables

We defined rural residence using 2003 Rural-Urban Continuum Codes (RUCCs) developed by the Economic Research Service of the US Department of Agriculture (eTable in the Supplement).^6^ We matched RUCCs with patient zip codes using 2010 United States Postal Service Zip Code Crosswalk Files from the US Department of Housing and Urban Development.^7^ Guided by recent studies in the literature, our primary analysis used a prespecified cut point, dividing the 9 RUCCs into 2 categories: urban (RUCCs 1-3) vs rural (RUCCs 4-9).^8,9,10^

Each analysis adjusted for the demographic variables age (<65 years vs ≥65 years), self-reported race (black vs other), and sex (where appropriate), which could potentially influence the relationship between residency status and survival outcomes. In addition, each analysis adjusted for important disease-specific clinical adjustment variables. Only clinical adjustment variables with a known effect on survival that were measured in all studies within an analysis were included (see Table 1 for clinical adjustment variables). These variables often represented those factors used to balance randomization assignment (ie, stratification factors).

**Table 1.** Study Descriptions

Type of Cancer	Trials by Study No.	Enrollment Date Range	Major Eligibility Criteria	Clinical Prognostic Factors^a^	Rural/Total Sample, No. (%)
Brain	S8737, S0001	1988-2005	No prior chemotherapy; performance status ≤2	Prior surgery: biopsy only vs resection; performance status: 0-1 vs >1	74/323 (22.9)
Breast, adjuvant ER-negative and PR-negative	S8897, S9313, S9623, S0012, S0221, S0307	1989-2012	Early stage (I-III); no prior chemotherapy for this breast cancer (for S0307, chemotherapy allowed concurrent with registration)	No. of positive lymph nodes: ≥4 vs <4; tumor size: ≤5 cm vs >5 cm; premenopausal vs postmenopausal	875/5026 (17.4)
Breast, adjuvant ER-positive and/or PR-positive	S8814, S8897, S9313, S9623, S0012, S0221, S0307	1989-2012	Early stage (I-III); no prior chemotherapy for this breast cancer (for S0307, chemotherapy allowed concurrent with registration)	No. of positive lymph nodes: ≥4 vs <4; tumor size: ≤5 cm vs >5 cm; premenopausal vs postmenopausal	1977/11 413 (17.3)
Breast, advanced	S0226, S0500	2004-2012	Stage IV; no prior chemotherapy for metastatic disease	*ERBB2* (formerly *HER2*)–negative vs *ERBB2*-positive; premenopausal vs postmenopausal; ER-positive and/or PR-positive vs ER-negative and PR-negative	244/1247 (19.6)
Colorectal, advanced	S8611, S8905, S9420	1987-1999	Metastatic (disseminated); maximum 1 prior adjuvant chemotherapy or immunotherapy	Performance status: 0-1 vs 2	286/1431 (20.0)
Gastric, adjuvant	S9008	1991-1998	Stages IB-IV (M0)	No. of involved lymph nodes: 0 vs 1-3 vs ≥4; T stage: T1-T2 vs T3-T4	83/488 (17.0)
Colorectal, adjuvant	S9304, S9415	1994-2000	Stage M0; no prior systemic or radiation therapy	No. of nodes involved: N0 vs N1 vs N2-N3; performance status: 0-1 vs 2; invasion of perirectal fat or adjacent organs: T1-T2 vs T3-T4	605/2593 (23.3)
Prostate 1, advanced	S8894	1989-1994	D2 disease	Performance status: <2 vs ≥2; severity of disease: minimal vs extensive	304/1333 (22.8)
Prostate 2, advanced	S9346	1995-2008	D2 disease	Performance status: <2 vs ≥2; severity of disease: minimal vs extensive	320/2055 (15.6)
Prostate, advanced hormone refractory	S9916, S0421	1999-2010	Metastatic prostate cancer that is unresponsive or refractory to hormone therapy; maximum 1 prior systemic therapy	Extraskeletal metastatic disease: yes vs no; PSA progression only: yes vs no; performance status: <2 vs ≥2	325/1658 (19.6)
Ovarian, advanced	S8501, S8790, S9701	1986-2001	Stage III or IV	Disease: optimal stage III vs suboptimal stage III or stage IV^b^	170/903 (18.8)
Acute myeloid leukemia	S8600, S8706, S9031, S9333, S0106	1986-2009	No previous systemic chemotherapy for acute leukemia	SWOG performance status: <2 vs ≥2	455/1748 (26.0)
NSCLC, advanced	S8738, S9308, S9509, S0003	1988-2002	No previous systemic chemotherapy or biologic therapy for NSCLC	Weight loss: <5% vs ≥5%; LDH: normal (≤ULN) vs abnormal (>ULN); stage of disease: IIIB vs IV	304/1461 (20.8)
Non-Hodgkin lymphoma, advanced aggressive	S8516, S9704	1986-2007	Intermediate or high-grade histology; no prior chemotherapy or radiation therapy for lymphoma^c^	IPI risk: low vs low-intermediate vs high-intermediate vs high	278/1155 (24.1)
Non-Hodgkin lymphoma, advanced indolent	S0016, S8809	1988-2008	Stages IIB-IV; no prior chemotherapy or radiation therapy for lymphoma	IPI risk: low vs low-intermediate vs high-intermediate vs high	209/1035 (20.2)
Myeloma, multiple	S8624, S9028, S9210, S9321, S0232, S0777	1987-2012	No prior chemotherapy for this disease	Stage: I-II vs III	568/2493 (22.8)
Gastrointestinal stromal tumor, advanced	S0033	2000-2001	Metastatic or unresectable	Performance status: 0-2 vs 3; measurable vs nonmeasurable disease	107/633 (16.9)
Total	44 unique trials	1986-2012			7184/36 995 (19.4)

Abbreviations: ER, estrogen receptor; IPI, International Prognostic Index; LDH, lactate dehydrogenase; NSCLC, non–small cell lung cancer; PR, progesterone receptor; PSA, prostate-specific antigen; ULN, upper limit of normal.

^a^ Including age (<65 vs ≥65 years), race (black vs other), and sex (where appropriate).

^b^ Definitions for suboptimal vs optimal stage III differed by study.

^c^ Study S9704 allows a single course of chemotherapy consisting of cyclophosphamide, doxorubicin, vincristine, and prednisone with or without rituximab.

Because men represented a very small fraction of the patients with breast cancer, male patients were excluded from the 3 breast cancer groups, and sex was not included as a covariate in these analyses. Analyses of non-Hodgkin lymphoma did not include age as a separate covariate because age is included in the International Prognostic Index.

The primary outcome was overall survival (OS), measured as days from study registration to death by any cause or, for those patients still alive, as days to last contact (censored). Patients lost to follow-up were also censored at their date of last contact. We also examined progression-free survival (PFS) and cancer-specific survival (CSS) as secondary outcomes. We defined PFS as days from study registration to the date of death by any cause, evidence of protocol-defined relapse or progressive disease, or date of last contact for those alive and progression free (censored). We measured CSS as days from study registration to date of cancer-specific death, death by another cause (censored), or last contact for those alive at last contact (censored). Detailed cause-of-death information was available for 28.0% of patient deaths. For the remaining 72.0%, we considered any death preceded by documented relapse or progression a cancer-specific death. We limited analyses to the first 5 years after registration in our main analysis in order to focus on cancer-related and treatment-related survival. Patients with last contact date or death date greater than 5 years postregistration were censored at 5 years of follow-up. Survival outcomes were grouped by adjuvant (ie, nonmetastatic) vs advanced (ie, metastatic) to identify whether global patterns of outcomes for rural vs urban patients differed by these disease settings.

### Statistical Analysis

To examine observed survival differences by residency status, we generated Kaplan-Meier OS curves for each of the 17 cancer cohorts, separately for rural and urban patients.^11^ We used multivariate Cox regression to estimate the effect of patient residence on survival outcomes while controlling for the major disease-specific prognostic factors and the demographic variables.^12^ Each analysis was stratified by study.

We further explored whether patterns of survival between rural and urban patients might substantively differ for different definitions of rural residency. We used variable cut point analysis to examine the association of residency and OS for each of the 8 possible definitions of rural residency based on the RUCCs.^13,14^ Although the primary analysis was based on 5 years of follow-up, for the variable cut point analysis, we allowed follow-up to vary from 1 to 10 years to allow for different prognosis patterns across the panel of 17 cancer types, resulting in 80 separate analyses per cancer type, or 1360 analyses overall. For each analysis, we derived the signed (according to the direction of the effect) χ^2^ statistic for the model-estimated association of rural or urban residence and OS in multivariable Cox regression.^12^ We treated these 17 χ^2^ statistics as independent random variables and used a 1-sample *t* test to assess whether they collectively differed from 0. We then plotted the corresponding signed *z* scores.

Tests for statistical significance were 2-sided, α = .05. Informal (*P* = .01) and formal (per Bonferroni) multiple comparison tests for the variable cut point analysis were provided. Data through January 30, 2018, were examined.

## Results

**Figure 1.**
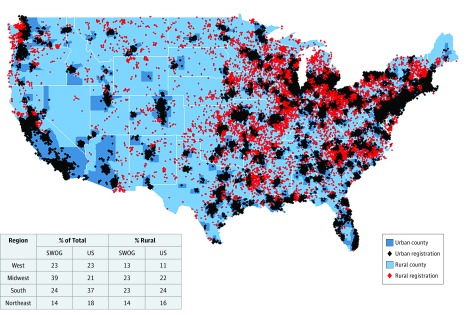
Map Showing 36 995 SWOG Enrollments From 1986 to 2012 by Rural vs Urban County Origin The percentage of total SWOG and US cancer population cases by region are shown in the table, along with the estimated proportion in rural areas for each region.

Overall, 36 995 patients enrolled between January 1, 1986, and December 31, 2012, in 44 SWOG phase 2/3 or phase 3 trials were analyzed (Table 1). Of the total study population, 19.4% resided in rural areas, the same as the rural proportion of the US population with cancer. Figure 1 shows a map of the trial registrations by rural vs urban status. Although all states are represented, the states with the fewest enrollments were Maine (82), Wyoming (93), and Rhode Island (102). Registrations in SWOG trials generally reflect the geographic population patterns of the United States, although with more patients enrolled from the Midwest (39% vs 21%) and fewer from the south (24% vs 37%). Rural patients were well represented within major geographic regions in the United States (West, Midwest, South, and Northeast) (Figure 1).

### Patient Characteristics

Overall, 27.7% (range across the 17 cohort analyses, 7.8%-74.5%) of patients (10 247 of 36 995) were aged 65 years or older, 40.3% (range across 17 cohort analyses, 28.1%-45.9%; 5381 of 13 360 patients) were female in the non-sex-specific analyses, and 10.8% (range across 17 cohort analyses, 1.9%-22.4%; 4003 of 36 995 patients) were black. Table 2 shows these statistics as well as descriptions of the clinical prognostic factors used in the analyses, both overall and separately by rural and urban residence. Patients residing in rural areas were older on average (rural, 30.7% aged ≥65 years vs urban, 27.0% aged ≥65 years; difference, 3.7%; 95% CI, 2.5%-4.9%; *P *< .001), were similar with respect to sex (rural, 40.4% female vs urban, 39.7% female; difference, 0.6%; 95% CI, −1.4% to 2.6%; *P *= .53), and were less likely to report being black (rural, 5.4% vs urban, 12.1%; difference, 6.7%; 95% CI, 6.1%-7.3%; *P *< .001) than patients residing in urban areas. There were very few statistically significant differences between rural and urban patients with respect to clinical prognostic factors.

**Table 2.** Patient Characteristics

Cancer Cohort	Patients, No. (%)									
	Overall	Demographic Factors			Clinical Prognostic Factors^b^					
		Age ≥65 y	Female^a^	Black	Factor 1		Factor 2		Factor 3	
**Brain**										
Overall	323 (100)	61 (18.9)	124 (38.4)	6 (1.9)	Performance status >1	69 (21.4)	Prior surgery biopsy only	79 (24.5)	NA	
Rural	74 (22.9)	18 (24.3)	28 (37.8)	1 (1.4)		16 (21.6)		18 (24.3)		
Urban	249 (77.1)	43 (17.3)	96 (38.6)	5 (2.0)		53 (21.3)		61 (24.5)		
**Breast, Adjuvant ER-Negative and PR-Negative**										
Overall	5026 (100)	394 (7.8)	NA	655 (13.0)	Tumor >5 cm	462 (9.2)	Postmenopausal	2402 (47.8)	≥4 Nodes^c^	581 (12.0)
Rural	875 (17.4)	84 (9.6)^d^		55 (6.3)		82 (9.4)		442 (50.5)		116 (13.6)
Urban	4151 (82.6)	310 (7.5)		600 (14.5)^e^		380 (9.2)		1960 (47.2)		465 (11.6)
**Breast, Adjuvant ER-Positive and/or PR-Positive**										
Overall	11413 (100)	1552 (13.6)	NA	822 (7.2)	Tumor >5 cm	957 (8.4)	Postmenopausal	6458 (56.6)	≥4 Nodes^c^	2504 (22.3)
Rural	1977 (17.3)	333 (16.8)^e^		59 (3.0)		162 (8.2)		1221 (61.8)^e^		433 (22.3)
Urban	9436 (82.7)	1219 (12.9)		763 (8.1)^e^		795 (8.4)		5237 (55.5)		2071 (22.3)
**Breast, Advanced**										
Overall	1247 (100)	473 (37.9)	NA	146 (11.7)	ER-negative and PR-negative	184 (14.8)	Postmenopausal	1134 (90.9)	*ERBB2* (formerly *HER2*)–negative^c^	978 (86.5)
Rural	244 (19.6)	113 (46.3)^e^		19 (7.8)		26 (10.7)		233 (95.5)^e^		187 (86.2)
Urban	1003 (80.4)	360 (35.9)		127 (12.7)^d^		158 (15.8)^d^		901 (89.8)		791 (86.5)
**Colorectal, Advanced**										
Overall	1431 (100)	594 (41.5)	566 (39.6)	189 (13.2)	Performance status = 2	143 (10.0)	NA		NA	
Rural	286 (20.0)	115 (40.2)	103 (36.0)	24 (8.4)		28 (9.8)				
Urban	1145 (80.0)	479 (41.8)	463 (40.4)	165 (14.4)^e^		115 (10.0)				
**Gastric, Adjuvant**										
Overall	488 (100)	164 (33.6)	137 (28.1)	84 (17.2)	Stage T3-T4	328 (67.2)	≥4 Nodes	205 (42.0)	NA	
Rural	83 (17.0)	34 (41.0)	16 (19.3)	7 (8.4)		60 (72.3)		41 (49.4)		
Urban	405 (83.0)	130 (32.1)	121 (29.9)	77 (19.0)^d^		268 (66.2)		164 (40.5)		
**Colorectal, Adjuvant**										
Overall	2593 (100)	988 (38.1)	1050 (40.5)	182 (7.0)	Performance status = 2	75 (2.9)	2-3 Nodes	803 (31.0)	Stage T3-T4	2212 (85.3)
Rural	605 (23.3)	234 (38.7)	253 (41.8)	9 (1.5)		14 (2.3)		187 (30.9)		520 (86.0)
Urban	1988 (76.7)	754 (37.9)	797 (40.1)	173 (8.7)^e^		61 (3.1)		616 (31.0)		1692 (85.1)
**Prostate 1, Advanced**										
Overall	1333 (100)	993 (74.5)	NA	298 (22.4)	Performance status ≥2	50 (3.8)	Severity = extensive	1057 (79.3)	NA	
Rural	304 (22.8)	230 (75.7)		39 (12.8)		10 (3.3)		239 (78.6)		
Urban	1029 (77.2)	763 (74.1)		259 (25.2)^e^		40 (3.9)		818 (79.5)		
**Prostate 2, Advanced**										
Overall	2055 (100)	1296 (63.1)	NA	384 (18.7)	Performance status ≥2	150 (7.3)	Severity = extensive	1470 (71.5)	NA	
Rural	320 (15.6)	201 (62.8)		28 (8.8)		22 (6.9)		223 (69.7)		
Urban	1735 (84.4)	1095 (63.1)		356 (20.5)^e^		128 (7.4)		1247 (71.9)		
**Prostate, Advanced Hormone Refractory**										
Overall	1658 (100)	1174 (70.8)	NA	229 (13.8)	Performance status ≥2	150 (9.0)	Extraskeletal metastases	879 (53.0)	Non-PSA progression	1345 (81.1)
Rural	325 (19.6)	246 (75.7)^d^		24 (7.4)		28 (8.6)		146 (44.9)		271 (83.4)
Urban	1333 (80.4)	928 (69.6)		205 (15.4)^e^		122 (9.2)		733 (55.0)^e^		1074 (80.6)
**Ovarian, Advanced**										
Overall	903 (100)	247 (27.4)	NA	27 (3.0)	Stage IV or suboptimal III	109 (12.1)	NA		NA	
Rural	170 (18.8)	50 (29.4)		5 (2.9)		21 (12.4)				
Urban	733 (81.2)	197 (26.9)		22 (3.0)		88 (12.0)				
**Acute Myeloid Leukemia**										
Overall	1748 (100)	341 (19.5)	803 (45.9)	155 (8.9)	Performance status ≥2	405 (23.2)	NA		NA	
Rural	455 (26.0)	101 (22.2)	216 (47.5)	25 (5.5)		112 (24.6)				
Urban	1293 (74.0)	240 (18.6)	587 (45.4)	130 (10.1)^e^		293 (22.7)				
**Non–Small Cell Lung Cancer, Advanced**										
Overall	1461 (100)	573 (39.2)	461 (31.6)	181 (12.4)	≥5% Weight loss	564 (38.6)	Abnormal LDH	591 (40.5)	Stage IV	1331 (91)
Rural	304 (20.8)	129 (42.4)	90 (29.6)	20 (6.6)		117 (38.5)		119 (39.1)		272 (89)
Urban	1157 (79.2)	444 (38.4)	371 (32.1)	161 (13.9)^e^		447 (38.6)		472 (40.8)		1059 (92)
**Non-Hodgkin Lymphoma, Advanced Aggressive**										
Overall	1155 (100)	237 (20.5)	483 (41.8)	106 (9.2)	High or high-intermediate IPI	568 (49.2)	NA		NA	
Rural	278 (24.1)	66 (23.7)	110 (39.6)	10 (3.6)		134 (48.2)				
Urban	877 (75.9)	171 (19.5)	373 (42.5)	96 (10.9)^e^		434 (49.5)				
**Non-Hodgkin Lymphoma, Advanced Indolent**										
Overall	1035 (100)	122 (11.8)	427 (41.3)	45 (4.3)	High or high-intermediate IPI	157 (15.2)	NA		NA	
Rural	209 (20.2)	26 (12.4)	95 (45.5)	8 (3.8)		41 (19.6)				
Urban	826 (79.8)	96 (11.6)	332 (40.2)	37 (4.5)		116 (14.0)				
**Myeloma, Multiple**										
Overall	2493 (100)	751 (30.1)	1047 (42.0)	423 (17.0)	Stage III	1462 (58.6)	NA		NA	
Rural	568 (22.8)	175 (30.8)	243 (42.8)	51 (9.0)		343 (60.4)				
Urban	1925 (77.2)	576 (29.9)	804 (41.8)	372 (19.3)^e^		1119 (58.1)				
**Gastrointestinal Stromal Tumor, Advanced**										
Overall	633 (100)	287 (45.3)	283 (44.7)	71 (11.2)	Performance status = 3	19 (3.0)	Measurable disease	599 (94.6)	NA	
Rural	107 (16.9)	49 (45.8)	44 (41.1)	5 (4.7)		5 (4.7)		102 (95.3)		
Urban	526 (83.1)	238 (45.3)	239 (45.4)	66 (12.5)^d^		14 (2.7)		497 (94.5)		
**Total**										
Overall	36 995 (100)	10 247 (27.7)	5381 (40.3)	4003 (10.8)	NA		NA		NA	
Rural	7184 (19.4)	2204 (30.7)^e^	1198 (40.4)	389 (5.4)						
Urban	29 811 (80.6)	8043 (27.0)	4183 (40.3)	3614 (12.1)^e^						

Abbreviations: ER, estrogen receptor; IPI, International Prognostic Index; LDH, lactate dehydrogenase; NA, not available; PR, progesterone receptor; PSA, prostate-specific antigen.

^a^ Percentage provided for the non-sex-specific cancers only.

^b^ Clinical prognostic factors are described in Table 1. The percentage of patients having each factor’s higher risk category is displayed in Table 2.

^c^ Percentage of patients with nonmissing data.

^d^ Statistically significantly higher, *P* < .05.

^e^ Statistically significantly higher, *P* < .01.

### Survival Outcomes

**Figure 2.**
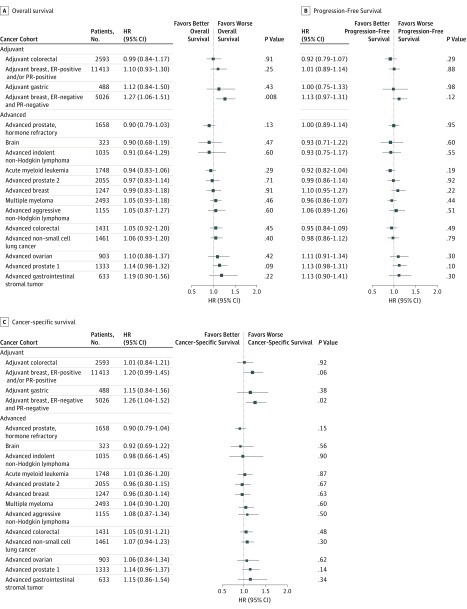
Forest Plot Showing the Association of Rural Residence and Survival Outcomes From Cox Regression Analyses Results are grouped by adjuvant vs advanced disease and ordered in ascending order of the overall survival hazard ratio (HR). Each horizontal bar represents the 95% confidence interval for the associated HR (box). Hazard ratios to the left of the line of equal hazard indicate better survival for rural patients, and HRs to the right of line of equal hazard indicate worse survival for rural patients. ER indicates estrogen receptor; PR, progesterone receptor.

The median follow-up time among patients still alive at the date of last contact for the primary analysis was the same for rural and urban patients (5.0 years each). There were few clear observed differences by residency status in OS using Kaplan-Meier curves (eFigure in the Supplement). In patients with adjuvant-stage estrogen receptor–negative, progesterone receptor–negative breast cancer, multivariate Cox regression results showed worse OS (hazard ratio, 1.27; 95% CI, 1.06-1.51; *P* = .008) and worse CSS (hazard ratio, 1.26; 95% CI, 1.04-1.52; *P* = .02) for patients residing in rural areas. No other statistically significant differences for any survival outcome for any of the remaining 16 disease cohorts were observed (Figure 2).

### Variable Cut Point Analyses

**Figure 3.**
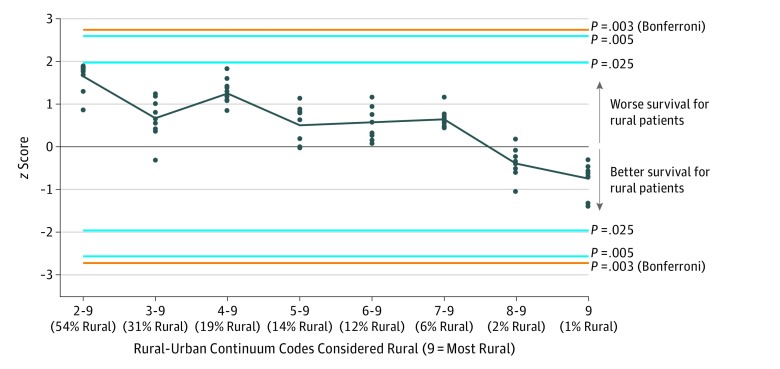
Association of Rural Residency and Overall Survival for Different Cut Points of Rural-Urban Continuum Codes The *z *scores (each represented by a circle) reflect the strength and direction of the association of residence and overall survival for each combination of Rural-Urban Continuum Code cut point (8 different cut points) and follow-up time (1-10 years) across the panel of 17 cancer cohorts. Negative *z *scores reflect better outcomes for rural patients; positive *z *scores reflect worse outcomes for rural patients. The dark gray line connects the mean values for each cut point. The blue horizontal lines show 2-tailed critical α levels for *P* = .05 and *P* = .01 (representing an informal adjustment for multiple comparisons), and the orange horizontal lines show 2-tailed critical α levels for *P* = .006 (representing a Bonferroni adjustment for multiple [n = 8] comparisons).

Figure 3 shows the results of the variable cut point analyses. Negative *z *scores reflect better outcomes for rural patients; positive *z *scores reflect worse outcomes for rural patients. The division of the RUCCs into urban (1) vs rural (2-9) maximized the association between residence and OS across the panel of 17 cancer cohorts. This categorization is problematic, however, because defining codes 2 and 3 as rural is not representative and results in a rural-to-urban ratio that is much different from that of the US population. The second highest average association between residence and OS occurred for rural defined as RUCCs 1 to 3 vs urban defined as RUCCs 4 to 9, reflecting the cut point specified for our primary analysis. Importantly, there was no evidence that any combination of RUCC cut point and follow-up time (1 to 10 years) resulted in a general pattern of better or worse survival for rural patients with cancer across the 17 cancer cohorts.

## Discussion

Numerous prior studies have documented inferior survival for rural patients with cancer.^10,15,16,17^ However, these studies did not adequately account for differences in access to cancer care. To our knowledge, no prior study has systematically compared cancer outcomes in clinical trial participants who reside in urban vs rural settings. This comprehensive analysis examined nearly 37 000 patients with a wide variety of cancer types and cancer stages. All participants were uniformly staged, treated, and followed up under protocol-directed care in a trial setting. Although rural trial participants were older on average and less likely to identify as black than their urban counterparts, after adjustment for these factors and important clinical prognostic factors, we found no systematic pattern of differences in survival outcomes by residency status, even under varied definitions of rural residency. Thus, this study contributes to the overall evidence base about differences in survival outcomes between rural and urban patients with cancer by showing that under similar treatment conditions within a clinical trial setting, rural and urban patients having the included cancer types do in fact have similar outcomes. This finding suggests that previously observed differences in outcomes for rural and urban patients with cancer may be due, in part, to inadequate receipt of guideline-concordant cancer care (as provided in clinical trials) rather than other factors intrinsic to residing in rural areas, such as unmeasurable differences in cancer prognosis or socioeconomic status.^18^ This conclusion is reinforced by the observation of very few differences in clinical prognostic factors between rural vs urban patients in our study.

It is more difficult to access adequate medical care for rural individuals.^3,19^ In the United States, rural residency has emerged as an important predictor of care access in the general population of individuals with cancer.^2^ At the same time, rural oncology resources are sparse; although 20% of the population is rural, only 3% of oncologists work in rural areas.^20^ Rural patients with cancer are required to travel much greater distances to receive care, adding time and financial burdens to treatment. One study found that fewer than 50% of rural patients with colorectal cancer in small or isolated rural areas had access to an oncologist within 30 miles.^21^ This travel burden can result in lower rates of standard care; rural patients with cancer were found to be about half as likely to receive breast-conserving therapy for early-stage breast cancer compared with the national average, and rates declined further as travel distance increased.^22^ A large study of nearly 35 000 patients found that patients with stage III colon cancer who had to travel very long distances (≥250 miles) to visit an oncologist were only one-third as likely to receive adjuvant chemotherapy.^23^ Research from Australia proposes numerous access issues that affect receipt of quality care, including delayed diagnosis due to limited access to screening or prevention tools, limited access to treating specialists, financial barriers to travel for treatment, and the physical burden of travel.^24,25,26^ Furthermore, residents of rural areas are least likely to have private health care coverage, accentuating disparities in access to health care and prevention services.^27,28^ Unfortunately, our study is unable to shed light on these issues given that all patients we studied were enrolled in trials.

These findings have implications for the current policy debates regarding the Affordable Care Act (ACA). Nineteen percent of patients with cancer reside in rural areas, the same as the rate of clinical trial patients from rural areas observed in this study.^1,2^ Therefore, rural patients with cancer make up a sizeable minority of patients with cancer who may have special needs.^29^ It is estimated that at least 22 million people will lose health care coverage if the ACA is dismantled, 14 million through loss of expanded Medicaid coverage.^30^ In many states, rural patients benefit disproportionately from Medicaid expansion. Adequate insurance is key to ensuring access to quality care. Therefore, reducing insurance access through policy prescriptions that limit or dismantle the ACA may disproportionately impact health care access for rural patients, again further widening disparities in outcomes between rural and urban patients with cancer.

Models for improving access to quality cancer care in rural settings do exist. In Australia, the establishment of Regional Cancer Centers of Excellence are linked to major urban cancer centers to provide multidisciplinary care, improve support services, and improve clinical trial participation.^31^ Successful centers have substantially increased the number of patients treated locally, removing a significant burden for rural patients. A shared approach between local practitioners and specialists could be a particularly useful model.^32^ Communications facilitated through teleoncology models have been found to improve access to specialist consultations and receipt of chemotherapy closer to home.^32,33^ In the United States, the NCI’s National Community Oncology Research Program, of which SWOG is a member, is designed to bring clinical trials to community investigators and patients.^34^ The success of this program is reflected by the results of our study, showing representative enrollment of rural patients with cancer from different regions throughout the United States (Figure 1). The Cancer Moonshot Blue Ribbon Report recommends the establishment of a large-scale patient participation network for comprehensive tumor profiling.^4^ Patients would enroll directly in the network, an approach that could reduce barriers to access to comprehensive cancer testing and novel treatments for rural and other medically disadvantaged groups.

### Limitations

The results of this analysis are limited by the fact that they may not represent patterns of outcomes for rural vs urban patients receiving quality care outside of a trial setting. Clinical trial participants have been shown to have better outcomes than those treated in a nontrial setting in the short term (ie, the first year after diagnosis), likely due to differences in baseline comorbid conditions.^35^ Although this could impact the absolute estimates of survival outcomes for patients examined in this analysis, it is less likely to affect the relative rates by rural vs urban status. Moreover, a randomized comparison of rural vs urban patients and cancer survival outcomes is clearly not feasible in this setting; thus, although our regression results accounted for important demographic and clinical prognosis factors, the potential remains that unknown confounders could influence the results. For instance, rural patients who participate in clinical trials may also be more likely to have improved health behaviors,^2^ although we found very little evidence of measurable differences between rural and urban patients in clinical risk factors (Table 2). Also, travel distance may lead to trial participation barriers that differ between rural and urban patients with cancer. However, the influence of this factor for patients choosing to participate in trials (as opposed to receiving care outside of a trial) is uncertain,^36^ especially because patients in urban areas may also choose to travel long distances for trial participation. The analysis of CSS was limited by incomplete cause-of-death information. Furthermore, we cannot presently explain why rural patients in the estrogen receptor–negative, progesterone receptor–negative, adjuvant breast cancer cohort had worse survival. One possibility is that residency is more relevant in adjuvant cancer settings, where prognosis is better overall, although we observed survival differences in only 1 of the 4 adjuvant cohorts we examined. Another possibility is that rural patients with cancer may be more likely to have delays in chemotherapy administration, which can adversely affect survival in patients with estrogen receptor–negative, progesterone receptor–negative cancers in particular.^37^

## Conclusions

Substantial efforts to identify and mitigate disparities in access to care and outcomes based on race, ethnicity, and socioeconomic factors have received great attention.^38^ Yet few efforts or research have focused on disparities related to geographic residency, despite the fact that the rural population in the United States represents 1 in 5 individuals. This is the largest examination of survival outcomes for rural vs urban patients with cancer receiving care in a clinical trial setting. The finding of almost no differences in outcomes by residency status across 17 different patient cohorts has potential policy implications. If rural and urban patients with cancer receiving similar care also have similar outcomes, then a reasonable inference is that the best means by which to improve outcomes for rural patients with cancer may be to improve their access to quality care. Better access to affordable health care insurance, better access to screening and prevention tools, better access to treating specialists, improved resources for traveling to receive care, and innovative new networks to give rural patients better access to new, novel treatments and clinical trials are all likely to improve outcomes for patients with cancer in rural areas. Thus multiple parallel efforts may begin to ameliorate the persistent disparity in cancer outcomes between rural and urban patients.

## Notes

Supplement. **eTable.** 2003 Rural-Urban Continuum Codes **eFigure.** Kaplan-Meier Survival Curves for Rural vs Urban Patients, in Order of Descending 2-Year Overall Survival Estimates for Urban Patients
