# Supplementary material for: Geographic Distribution and Survival Outcomes for Rural Patients With Cancer Treated in Clinical Trials
Source: JAMA Netw Open. 2018 Aug 17;1(4):e181235. doi: 10.1001/jamanetworkopen.2018.1235 (PMC6324281; doi:10.1001/jamanetworkopen.2018.1235)
Supplement: Supplement. — eTable. 2003 Rural-Urban Continuum Codes eFigure. Kaplan-Meier Survival Curves for Rural vs Urban Patients, in Order of Descending 2-Year Overall Survival Estimates for Urban Patients [file jamanetwopen-1-e181235-s001.pdf]

Supplementary Online Content

Unger JM, Moseley A, Symington B, Chavez-MacGregor M, Ramsey SD, Hershman DL. Geographic Distribution and Survival Outcomes for Rural Patients With Cancer Treated in Clinical Trials. *JAMA Netw Open*. 2018;1(4):e181235. doi:10.1001/jamanetworkopen.2018.1235

eTable. 2003 Rural-Urban Continuum Codes
eFigure. Kaplan-Meier Survival Curves for Rural vs Urban Patients, in Order of Descending 2-Year Overall Survival Estimates for Urban Patients

This supplementary material has been provided by the authors to give readers additional information about their work.

© 2018 Unger JM et al. *JAMA Network Open.*

eTable. 2003 Rural-Urban Continuum Codes

Code	Description
**Metro counties:**	
1	Counties in metro areas of 1 million population or more
2	Counties in metro areas of 250,000 to 1 million population
3	Counties in metro areas of fewer than 250,000 population
**Nonmetro counties:**	
4	Urban population of 20,000 or more, adjacent to a metro area
5	Urban population of 20,000 or more, not adjacent to a metro area
6	Urban population of 2,500 to 19,999, adjacent to a metro area
7	Urban population of 2,500 to 19,999, not adjacent to a metro area
8	Completely rural or less than 2,500 urban population, adjacent to a metro area
9	Completely rural or less than 2,500 urban population, not adjacent to a metro area

© 2018 Unger JM et al. *JAMA Network Open.*

eFigure: Kaplan-Meier Survival Curves for Rural vs Urban Patients, in Order of Descending 2-Year Overall Survival Estimates for Urban Patients.

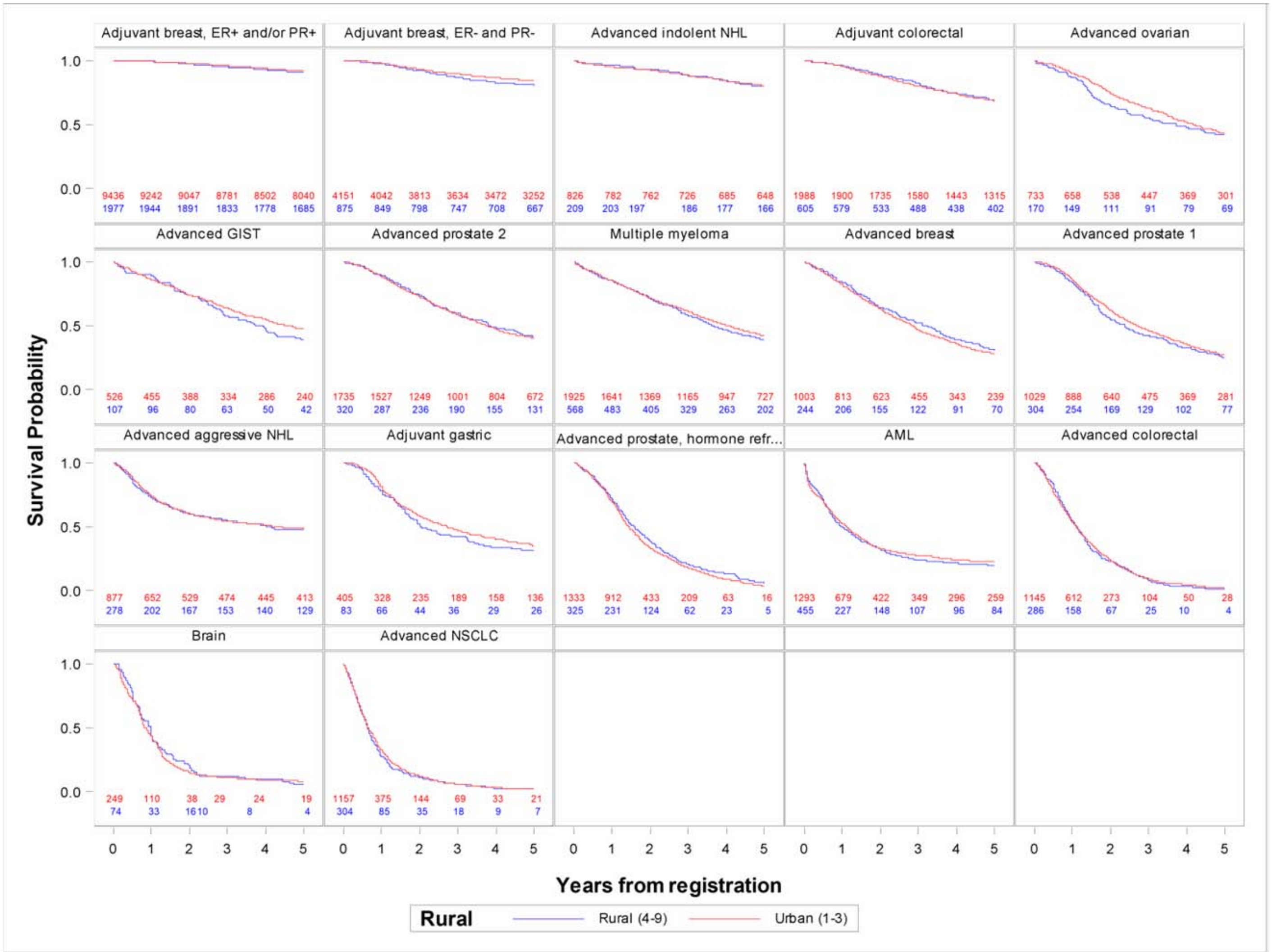

Numbers below figures show number at for rural (blue) vs. urban (red) groups. Rural (4-9) indicates non-metropolitan counties, and Urban (1-3) indicates metropolitan counties (see eTable 1). There is no clear pattern of differences by residence in overall survival. ER-, estrogen-receptor negative; PR-, progesterone-receptor negative; ER+, estrogen-receptor positive; PR+, progesterone-receptor positive; ; NHL, non-Hodgkin's lymphoma; GIST, gastro-intestinal stromal tumor; HR, hormone refractory; AML, acute myeloid leukemia; NCSCL, non-small cell lung cancer.
